# Evaluation of the predictive value of echocardiography parameters for heart transplant rejection: A tissue Doppler imaging observational study

**DOI:** 10.1016/j.ahjo.2025.100654

**Published:** 2025-10-24

**Authors:** Fereshteh Ghaderi, Hoorak Poorzand, Farveh Vakilian, Hedieh Alimi, Leila Bigdelu, Afsoon Fazlinezhad, Amirhossein Rafighdoost, Faeze Keihanian

**Affiliations:** a Department of Cardiovascular Diseases, Faculty of Medicine, Mashhad University of Medical Sciences, Mashhad, Iran; b Cardiovascular Division, Vascular and Endovascular Surgery Research Center, Mashhad University of Medical Sciences, Mashhad, Iran

**Keywords:** Heart transplant, Echocardiography, Transplant rejection, Endomyocardial biopsy

## Abstract

**Introduction:**

The rate of heart transplantation is increasing worldwide. Due to the limitations of endomyocardial biopsy (EMB), various non-invasive methods have been suggested to assess rejection. Therefore, the aim of this study was to evaluate the predictive value of echocardiographic parameters to determine heart transplant rejection in a sample of Iranian patients.

**Methods:**

This was a cross-sectional study on heart transplant patients with available EMB results. All patients underwent echocardiography at the same day of EMB and prior to biopsy. The association between echocardiographic parameters and rejection was assessed using binary logistic regression.

**Results:**

A total of 67 patients (50, 74.6 % male and 17, 25.4 % female) with the mean age of 39.20 ± 11.39 years were enrolled in this study. Heart transplant rejection was observed in 22 (32.8 %) patients. There was only a significant difference in mitral inflow peak early diastolic velocity (E) and septal time to peak systolic velocity in ejection phase (septal Ts) between rejection and non-rejection groups. Logistic regression revealed a significant association between rejection and septal Ts (*p* = 0.048, OR = 0.931) and E velocity (*p* = 0.022, OR = 78.069). Based on ROC curve, the area under the curve for septal Ts and E were 81.9 % and 68.6 %. Moreover, the sensitivity and specificity for septal Ts and E in detection of rejection were 75 %, 69 % and 68 %, 61 %, respectively.

**Conclusion:**

Septal Ts could be used as a valuable echocardiographic parameter for predicting rejection in heart transplant recipients.

## 1 Introduction

The rate of heart transplantation has increased in Iran since the first one performed successfully in 1992 [1]. The reported number of heart transplant in Iran has raised from 104 patients in 2008 to 122 ones in 2011 [2]. Similar to other organ transplantations, rejection is an important complication. Transplant rejection can be hyper-acute (between minutes and hours after transplant), which occurs due to the existence of antibodies against the graft in the host body before surgery, acute (between weeks to years after transplant), which can be due to cellular or humoral mechanism, and chronic rejection that may occur years after surgery [3]. Early diagnosis and management of acute cellular rejection (ACR) can lead to better outcomes and preserve heart function. Hence, annual rejection surveillance in most heart transplant centers have been performed for the first 5 years post-transplant, by endomyocardial biopsy (EMB) as the gold standard [4]. EMB is an invasive and expensive procedure. Additionally, it puts patients at risk for several serious complications, including pericardial tamponade and severe tricuspid regurgitation [5], third-degree atrioventricular block, trauma to vasculature and deep vein thrombosis [6]. Moreover it is not very widely used due to its long duration of sample preparation and evaluation, possibility of false negative results and a patchy rejection pattern [7]. ACR was reported in 34.4 % of transplants in one study in Iran [8], which is higher than the previously reported 25 % rejection in Europe [9]. The signs and symptoms of allograft heart rejection are usually non-specific therefore, diagnosis mainly done when heart failure is developed [3].

Unfortunately, lack of a suitable non-invasive modality for detection of heart transplant rejection has made it difficult. One of the non-invasive hypothetically beneficial techniques for early diagnosis of heart transplant rejection is tissue Doppler imaging (TDI) [10,11].

It was shown that the combination of two new echocardiographic measures, global LV and RV free wall longitudinal strain, may be able to identify a group of heart transplant patients who are unlikely to have ACR [12]. In a study on 64 ACR patients, global longitudinal strain using two-dimensional speckle-tracking echocardiography, was found to be reduced in moderate rejection and retained to normal values after the treatment. Furthermore, the E/A and E/e′ ratio were also found to be higher in patients with rejection [13]. In another study, a significant association was found between E wave and mild to moderate transplant rejection [14].

According to rates of heart transplant rejection in Iran and difficulties in performing routine EMB, as well as the availability of echocardiographic devices and experts in most hospitals in Iran, we aimed to assess the predictive value of TDI parameters in early detection of heart transplant rejection.

## 2 Methods

### 2.1 Subjects

This was a cross-sectional study including adult recipients who underwent EMB in Imam Reza Hospital, Mashhad, Iran from November 2018 to October 2020. A single team performed all the transplantations using the bicaval method. Demographic data including age and gender were extracted from hospital records.

### 2.2 Echocardiography

All patients underwent conventional echocardiography including TDI using the Philips iE33 scanner and S5-1 probe (Philips Healthcare, Bothell, WA, USA) at the same day the EMB was obtained. If there were signs or symptoms of heart failure, the patient was excluded.

### 2.3 Echocardiographic parameters

Echocardiographic parameters included left-ventricular end-diastolic diameter (LVEDD), left-ventricular end-systolic diameter (LVESD), interventricular septum thickness at end-diastole (IVSd), left atrial volume, right atrial area, left ventricular mass index (LVMI), myocardial peak systolic tissue velocity (Sm), myocardial early diastolic tissue velocity (Em) and left ventricular ejection fraction (LVEF). We also assessed myocardial peak late diastolic tissue velocity (Am), mitral inflow peak early diastolic (E) and late diastolic velocity (A), E/Em and E/Am ratios, deceleration time of mitral E wave (DT), isovolumic relaxation time (IVRT), septal time to peak systolic velocity in ejection phase (Septal Ts), segmental thickness variability (STV) and pulmonary artery pressure (PAP).

### 2.4 Endomyocardial biopsy

EMB was obtained by the heart transplant cardiologist according to the hospital post-surgery EMB protocol or at the time of clinical suspicion to heart transplant rejection. EMB specimens were achieved through jugular or femoral access by obtaining at least 5 endomyocardial samples from the right ventricular septum. Samples were prepared and analyzed by an expert pathologist based on histology and immunofluorescence techniques. Cellular rejection was graded from 0, indicating no rejection, to 2 based on the International Society of Heart and Lung Transplantation (ISHLT) 2005 grading system [13]. In this investigation, all patients with a rejection had a grade 1 pathological study. Antibody mediated rejection was also assessed. In this study, rejection was defined as any grade of cellular rejection (grade ≥ 1) or presence of antibody related characteristics.

### 2.5 Ethics

A written informed consent was obtained from all patients in both case and control groups prior to participation in the study. The study steps were performed in line with Helsinki declaration. Also, the study protocol was approved by the Ethical Committee of Mashhad University of Medical Sciences (Ethical ID: IR.MUMS.MEDICAL.REC.1398.222).

### 2.6 Statistical analysis

Data was analyzed using the statistical package for social sciences (SPSS) software version 21 (IBM Inc., Chicago, Il, USA). Normal distribution of continuous variables was assessed using the Shapiro-Wilk test. Continuous variables were presented using mean and standard deviation (SD), while categorical variables were presented using frequency and percentage. Mann-Whitney test was used to compare continuous variables between the study groups. Chi-square test was used to compare the distribution pattern of categorical variables. Backward binary logistic regression was also performed to assess the predictors of heart transplant rejection. The receiver operating characteristic (ROC) curve was determined to identify the cut-off values for predictors of heart transplant rejection. The level of statistical significance was set as *p* < 0.05.

## 3 Results

A total of 67 patients (50, 74.6 % male and 17, 25.4 % female) entered the study. The mean ± SD age was 39.20 ± 11.39 years with no significant difference between two groups. The echocardiographic findings of participants are presented in Table 1. Of 67 patients, transplant rejection was documented in 22 (32.8 %) ones. A comparison of study parameters between rejection and non-rejection groups is presented in Table 2. The values for E velocity was significantly higher in rejection group (*P* = 0.014), while septal TS values were significantly higher in non-rejection group (*P* < 0.0001). However, there were no significant differences in terms of other parameters between the groups (*P* > 0.05) (Table 1).

**Table 1:** Comparison of echocardiographic parameters between groups

Variable	Total		Non-rejection		Rejection		p
	Mean	SD	Mean	SD	Mean	SD	
LVEDD (cm)	4.44	0.62	4.46	0.46	4.40	0.87	0.789
LVESD (cm)	3.01	0.52	2.99	0.46	3.05	0.62	0.362
IVSd (cm)	0.84	0.09	0.85	0.08	0.84	0.11	0.947
LVEF (%)	54.97	8.97	54.23	9.66	56.63	7.11	0.409
LA volume (mL)	66.23	21.77	21.07	4.69	22.21	5.12	0.387
RA area (cm^2^)	15.64	8.41	15.54	1.00	15.83	4.34	0.091
LVMI (gr/m^2^)	105.02	16.06	104.53	17.84	105.90	12.56	0.611
Sm (cm/s)	6.09	1.38	6.22	1.51	5.82	1.08	0.108
Em (cm/s)	7.56	2.18	7.22	1.93	8.24	2.54	0.192
Am (cm/s)	5.71	1.56	5.80	1.52	5.54	1.65	0.361
E (m/s)	0.76	0.55	0.75	0.66	0.76	0.17	0.014⁎
A (m/s)	0.55	0.77	0.59	0.95	0.46	0.12	0.664
E/A	1.59	0.51	1.51	0.44	1.76	0.62	0.069
DT (ms)	148.78	45.16	153.03	41.21	140.95	51.93	0.370
IVRT (ms)	95.30	23.38	93.37	22.96	98.72	24.38	0.357
GLS (%)	−15.50	2.22	−16.00	1.55	−14.76	2.84	0.198
Septal Ts (ms)	92.49	23.17	101.28	16.87	75.35	24.54	<0.0001^⁎^
Mid RV diameter (cm)	3.16	0.37	3.16	0.40	3.17	0.28	0.672
STV (%)	8.59	1.36	8.69	1.50	8.40	1.04	0.654
PAP (mm Hg)	27.35	5.11	27.42	5.14	27.22	5.21	0.937

SD: Standard Deviation, LVEDD: left-ventricular end-diastolic diameter, LVESD: left-ventricular end-systolic diameter, IVSd: Interventricular septum thickness at end-diastole, LVEF: Left Ventricular Ejection Fraction, LA: Left Atrium, RA: Right Atrium, LVMI: left ventricular mass index, Sm: myocardial peak systolic tissue velocity, Em: myocardial early diastolic tissue velocity, Am: myocardial late diastolic tissue velocity, E: mitral peak early diastolic velocity, A: mitral peak late diastolic velocity, DT: deceleration time of mitral E wave, IVRT: isovolumic relaxation time, GLS: global longitudinal strain, Septal Ts: septal time to peak systolic velocity in ejection phase, Mid RV: longitudinal strain at mid right ventricle, STV: segmental thickness variability, PAP: Pulmonary Artery Pressure. The Mann-Whitney test was used for the comparison.

⁎ Significant difference between groups.

**Table 2:** Regression analysis to identify the variables that were significantly related to transplant rejection.

	p	OR	95 % C.I. for OR	
			Lower	Upper
E velocity	0.022⁎	78.069	1.871	3257.226
Septal TS	0.048⁎	0.931	0.867	0.970

E velocity: mitral peak early diastolic velocity, Septal TS: septal time to peak systolic velocity in ejection phase.

⁎ Significant at α = 0.05.

**Fig. 1:**
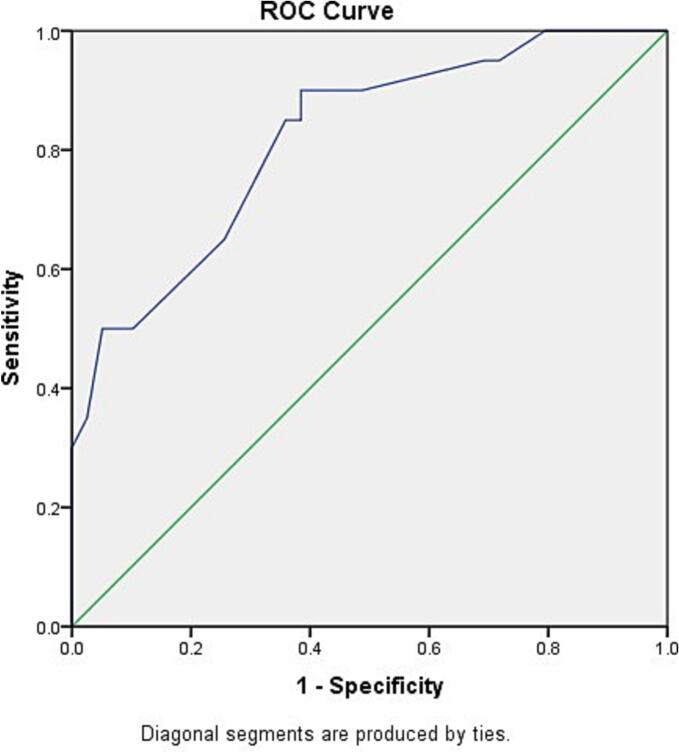
The ROC curve analysis to identify the cut-off point for septal TS to identify transplant rejection. To plot the ROC curve, septal TS values were subtracted from 126.

**Fig. 2:**
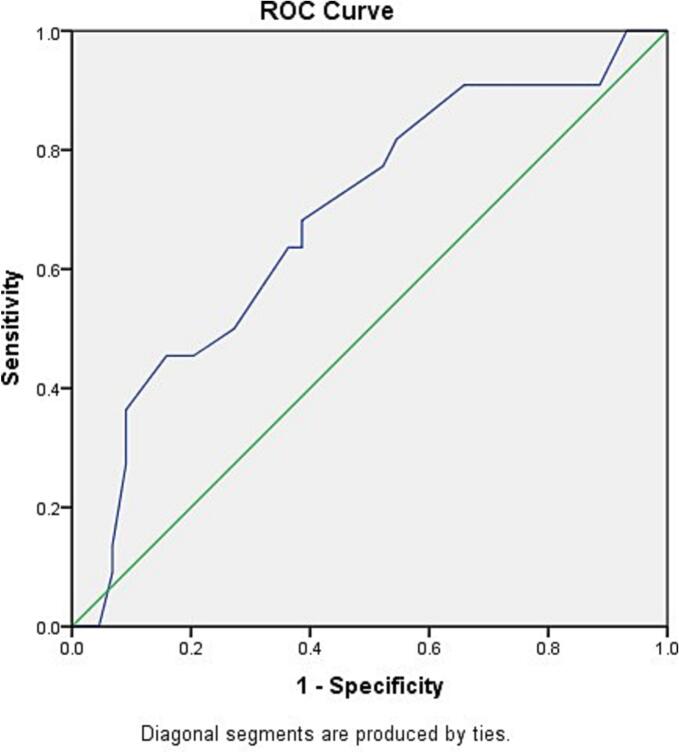
The ROC curve analysis to identify the cut-off point for E velocity to identify transplant rejection.

The regression analysis revealed that heart transplant rejection was related to E velocity (*P* = 0.022) and septal TS (*P* = 0.048) (Table 2). The plotted ROC curve was reverse for septal Ts, therefore to obtain a positive result for ROC curve, the values were subtracted from 126, which was larger than the maximum value of Septal TS in the study. The area under the curve (AUC) in reversed ROC curve for septal Ts was 81.9 %, which was statistically significant (*P* < 0.0001). The optimal cut-off for the transformed septal Ts values (126 - original value) was 33.5, When back-transformed, this corresponds to an original septal Ts value of 92.5. Therefore, original septal Ts values smaller than 92.5 were predictive of rejection (sensitivity 75 %, specificity 69 %) (Fig. 1). The ROC curve for E velocity had an AUC of 68.6 %, which was statistically significant (*P* = 0.014), but considered low based on the desired value of higher than 80 %. The cut-off value for E velocity was determined to be 0.65 with sensitivity of 68 % and specificity of 61 %. (Fig. 2).

Based on the reported ROC, septal Ts was a better predictor for heart transplant rejection. Therefore, a cross-tabulation was performed to demonstrate the validity of the test (Table 3). Categorization of study patients based on the identified cut-off value resulted in correctly identification of 77.3 % of rejection group patients and 60 % of non-rejection ones. A combination assessment tool was then designed considering septal Ts below 92.5 msec and E velocity above 0.65 as positive criteria and septal Ts above 92.5 msec and E velocity below 0.65 as negative criteria for rejection. In this combined tool, one or two positive criteria for rejection was considered positive and presence of two negative criteria was considered as negative. This combined tool resulted in the sensitivity and specificity of 90.0 % and 42.1 %, respectively.

**Table 3:** Categorization of study subjects based on the defined cut-off value.

		Rejection		p
		No rejection	Rejection	
Septal Ts	Positive (<92.5)	18 (40.0 %)	17 (77.3 %)	0.004⁎⁎
	Negative (≥92.5)	27 (60.0 %)	5 (22.7 %)	
E velocity	Positive (≥0.65)	17 (38.6 %)	15 (68.2 %)	0.024⁎
	Negative (<0.65)	27 (61.4 %)	7 (31.8 %)	
Combination	≥1 positive criteria	22 (57.9 %)	18 (90.0 %)	0.012⁎
	No positive criteria	16 (42.1 %)	2 (10.0 %)	

E velocity: mitral inflow early diastolic flow, Septal TS: Septal Ts: septal time to peak systolic velocity in ejection phase.

⁎ Significant at α = 0.05.

⁎⁎ Significant at α = 0.01.

## 4 Discussion

Echocardiography is among the non-invasive techniques for prediction of heart transplant rejection [12,15]. Reduction in LV mass and thickness as well as reduced systolic function and pericardial effusion are significant echocardiographic findings in heart transplant rejection, but these findings are not predictors due to a late onset [16]. In contrast to systolic dysfunction, diastolic dysfunction appears earlier in heart transplant rejection. However, because of the difference in the definition of diastolic function parameters, the use of these parameters have shown different accuracy in diagnosis of heart transplant rejection [17,18]. Furthermore, diastolic function might be affected by conditions including hypertension and allograft vasculopathy, which make the interpretation of the findings difficult [12,16,19,34]. Among the new measurements, the strain assessment by TDI has shown promising findings in early detection of heart transplant rejection [[20], [21], [22], [23]].

In this study heart transplant rejection was observed in 32.8 % of patients. This is in line with the findings of a previous study in Iran, which reported a 34.4 % rejection rate in heart transplant patients [8]. In contrast, the rate of rejection was lower in the reports of Europe (25 %) and the United Stated (7.8 %) [9,24]. One reason for the observed difference might be due to the younger age of patients in Iran compared to developed countries, as previously demonstrated that younger age was associated with higher risk for transplant rejection [18]. Another reason might be related to the differences in sample size and the number of EMBs performed based on different protocols.

This study demonstrated that E was the only significant factor that was higher in non-rejection group compared with the rejection group, the finding that showed in previous study, as well [18]. Unalike the findings of our study, Sun et al. revealed a significant difference in IVRT between rejection and non-rejection groups, but they also proved that IVRT might not be a good predictor for rejection based on the small sensitivity (53 %). They reported a restrictive mitral inflow pattern among patients with rejection [18]. This is in contrast with the study by Haghighi that reported no significant difference in TDI parameters between rejection and non-rejection groups [14]. It was stated that a restrictive pattern might be a specific finding in heart transplant rejection but it could potentially not have adequate sensitivity [14]. In another study, an association was found between posterior wall thickness and ACR of any grade in univariate analysis, but did not remain after multivariate analysis. They also found some associations of ACR with mitral inflow velocities determined by conventional pulsed Doppler, only in univariate analysis, that disappeared after adjusting by other parameters [25].

Another possible reason for such differences might be due to the method used for heart transplant. It was previously shown that heart transplantation using bicaval method might result in improved atrial function, while older techniques might lead to varying A velocity and thus increasing the E/A ratio which can resemble a restrictive pattern even in patients with no rejection [26]. However, all studies did not report any significant differences regarding E/A ratio between rejection and non-rejection groups, which is consistent with the findings of the current study [12,27].

Dandel et al. found that peak systolic and early diastolic peak wall motion velocity obtained at the basal LV posterior wall were significantly associated with rejection [17]. Puleo et al. reported similar results only for diastolic velocities [28]. Mankad et al. described that a peak-to-peak mitral annular velocity (the same s′ + e′ parameter) > 13.5 mm/s, determined by color-coded tissue Doppler, had 93 % sensitivity, 71 % specificity, and 98 % negative predictive value for detecting rejection, defined as grade IB or above of the former classification of the International Society of Heart and Lung Transplantation (ISHLT) [29]. Logistic regression also revealed that E was significantly related to rejection; one-unit increase in E was associated with 78.07 times increased risk for developing heart transplant rejection. Although this was significant, the wide CI for OR indicates that E may not be a sensitive predictor for heart transplant rejection. One reason for the various observations regarding the association of E or E/A velocity and the risk of heart transplant rejection might be due to the patchy nature of cellular rejection, which could result in different effects on flow and wall motion. It was also shown that E/A ratio remained unchanged after heart transplantation regardless of the normalization of diastolic function, which might also interfere with the interpretation of the observed patterns in heart transplant rejection [13]. In another study on heart transplant patients (723 echocardiographic assessments), significant variations were observed in DT, EF, E and E/A [30]. In the current study, the mean E velocity was lower compared to other studies that might have resulted in lower E/A values leading to a non-significant reduction in E/A ratio in the rejection group.

The findings of the current study revealed that septal Ts was significantly higher in non-rejection group compared to the rejection one. The relation between septal Ts and rejection was also documented in the current study after performing the logistic regression analysis. The regression analysis revealed that higher septal Ts was associated with 93.1 % reduced risk for heart transplant rejection. It was previously reported that heart transplantation may result in abnormal septal motion due to alterations in right ventricular pressure and conduction abnormalities [31]. This finding may either strengthen or weaken the diagnostic properties of septal Ts in detection of heart transplant rejection.

The ROC curve revealed that septal Ts could better detect rejection compared to E. The sensitivity and specificity for septal Ts for detection of rejection were 75 % and 69 % respectively, while the sensitivity and specificity for E velocity in detection of rejection were 65 % and 61 % respectively. Combination of septal Ts and E velocity increased the sensitivity to 90 %, but reduced the specificity to 42.1 %. Therefore, assessment of E velocity may be used in suspicious cases for rejection to double confirm the prediction but may not be useful in ruling out rejection in low suspicion cases based on septal Ts.

Septal Ts refers to the time interval (in milliseconds) from the onset of the QRS complex on the ECG to the peak systolic velocity of myocardial contraction in the sampled septal segment, measured using TDI. This parameter reflects regional myocardial mechanical activation and contractile function [32]. In clinical protocols, the septal region is chosen for consistency, reproducibility, and its central role in overall ventricular function. However, septal measurements may still miss rejection patches localized elsewhere in the myocardium, as demonstrated by the modest sensitivity and specificity found in the literature (e.g., septal Ts sensitivity 75 %, specificity 69 %). Sampling only a portion of the septum, as in standard practice, means the result is still prone to under-representing patchy involvement [33]. While septal Ts offers valuable insight into regional systolic function and may change with rejection, it does not overcome the central limitation of spatial sampling inherent to echocardiography. Multiple studies support that a truly global or more comprehensive assessment (such as averaging multiple segmental velocities or combining with other imaging modalities) may be required to enhance diagnostic sensitivity for patchy transplant rejection.

One of the limitations of this study was the sample size, which prevented us from performing subgroup analyses. Although the primary objective of the study was met, further studies are required to assess the effect of the type of transplant surgery technique and different grades of rejection on the sensitivity and specificity of the echocardiographic parameters. There is also a need for inclusion of older patients as the observed rejection pattern might be different in the elderly.

## 5 Conclusion

Septal Ts and the E velocity might play a role in early detection of heart transplant rejection but there is a need for larger studies to recommend echocardiographic parameters for follow-up of heart transplant patients.

## CRediT authorship contribution statement

**Fereshteh Ghaderi:** Writing – review & editing, Methodology, Investigation, Data curation, Conceptualization. **Hoorak Poorzand:** Validation, Data curation. **Farveh Vakilian:** Validation, Supervision, Resources, Conceptualization. **Hedieh Alimi:** Validation, Supervision. **Leila Bigdelu:** Validation, Supervision. **Afsoon Fazlinezhad:** Validation, Supervision. **Amirhossein Rafighdoost:** Writing – original draft, Investigation. **Faeze Keihanian:** Writing – review & editing, Writing – original draft, Resources, Data curation.

## Consent for publication

Written consent to publish this information was obtained from study participant.

## Ethical statement

The informed consent was obtained for experimentation with human subjects. This study carried out in accordance with The Code of Ethics of the World Medical Association (Declaration of Helsinki) for experiments involving humans.

## Ethics approval and consent to participate

A written informed consent was obtained from all patients in both case and control groups prior to participation in the study. The study steps were performed in line with Helsinki declaration. The study protocol was approved by the Ethical Committee of Mashhad University of Medical Sciences (Ethical ID: IR.MUMS.MEDICAL.REC.1398.222).

## Funding

This study was funded by 10.13039/501100004748Mashhad University of Medical Sciences.

## Declaration of competing interest

The authors declare that they have no known competing financial interests or personal relationships that could have appeared to influence the work reported in this paper.

## Data Availability

The data that support the findings of this study are available from Imam Reza Hospital, Mashhad University of Medical Sciences, but restrictions apply to the availability of these data, which were used under license for the current study, and so are not publicly available. Data are however available from the authors upon reasonable request and with permission of the corresponding author.
